# Cross-shift changes in pulmonary function and occupational exposure to particulate matter among e-waste workers in Ghana

**DOI:** 10.3389/fpubh.2024.1368112

**Published:** 2024-05-09

**Authors:** Zoey Laskaris, Marie S. O'Neill, Stuart A. Batterman, Bhramar Mukherjee, Julius N. Fobil, Thomas G. Robins

**Affiliations:** ^1^Department of Epidemiology, University of Michigan School of Public Health, Ann Arbor, MI, United States; ^2^Department of Epidemiology, Department of Environmental Health Sciences, University of Michigan School of Public Health, Ann Arbor, MI, United States; ^3^Department of Environmental Health Sciences, University of Michigan School of Public Health, Ann Arbor, MI, United States; ^4^Department of Biostatistics, University of Michigan School of Public Health, Ann Arbor, MI, United States; ^5^Department of Biological, Environmental, and Occupational Health Sciences, University of Ghana School of Public Health, Accra, Ghana

**Keywords:** electronic-waste, respiratory health, informal sector, air pollution, particulate matter, personal inhalation, pulmonary function, Ghana

## Abstract

**Introduction:**

Little is known on the association between cross-shift changes in pulmonary function and personal inhalation exposure to particulate matter (PM) among informal electronic-waste (e-waste) recovery workers who have substantial occupational exposure to airborne pollutants from burning e-waste.

**Methods:**

Using a cross-shift design, pre- and post-shift pulmonary function assessments and accompanying personal inhalation exposure to PM (sizes <1, <2.5 μm, and the coarse fraction, 2.5–10 μm in aerodynamic diameter) were measured among e-waste workers (*n* = 142) at the Agbogbloshie e-waste site and a comparison population (*n* = 65) in Accra, Ghana during 2017 and 2018. Linear mixed models estimated associations between percent changes in pulmonary function and personal PM.

**Results:**

Declines in forced expiratory volume in one second (FEV1) and forced vital capacity (FVC) per hour were not significantly associated with increases in PM (all sizes) among either study population, despite breathing zone concentrations of PM (all sizes) that exceeded health-based guidelines in both populations. E-waste workers who worked “yesterday” did, however, have larger cross-shift declines in FVC [−2.4% (95%CI: −4.04%, −0.81%)] in comparison to those who did not work “yesterday,” suggesting a possible role of cumulative exposure.

**Discussion:**

Overall, short-term respiratory-related health effects related to PM exposure among e-waste workers were not seen in this sample. Selection bias due to the “healthy worker” effect, short shift duration, and inability to capture a true “pre-shift” pulmonary function test among workers who live at the worksite may explain results and suggest the need to adapt cross-shift studies for informal settings.

## 1 Introduction

Reductions in occupational and environmental health risks associated with the recovery of valuable metals and plastics from used electronic and electrical equipment waste (e-waste) are urgently needed around the globe (1, 2). In informal e-waste recycling sectors common in low- and middle-income (LMIC) countries (e.g., Nigeria, Ghana, Thailand, Argentina), occupational and environmental health and safety regulations are often unenforced, putting workers and nearby communities at risk of exposure to a multitude of physical and chemical pollutants (3–5). Despite a growing body of evidence documenting the occupational and community-level health effects of exposure to e-waste associated pollutants [e.g., lead, chromium, cadmium, flame retardants, dioxins, furans, polycyclic aromatic hydrocarbons (PAHs), particulate matter (PM)] (6–8), little is known about the respiratory health effects among workers associated with air pollution generated from e-waste recovery practices (9–14).

Burning e-waste in open surface fires is a commonly used technique for efficiently eliminating plastic coatings from valuable metals in the informal sector. Measures of PM air pollution from burning e-waste can reach high concentrations (>500 μg m^−3^) and the PM can be comprised of a high fraction of toxic constituents (e.g., heavy metals, PAHs and flame retardants), posing risks to workers and surrounding communities (5, 10, 11, 13, 15–20). Other techniques used to process e-waste, such as manual dismantling of generators, cathode ray tubes, and fluorescent lighting, for example, present additional inhalation hazards including metal-contaminated dust and vapors (21).

Few occupational studies have measured acute responses to PM. Using data from wildland firefighters, Gaughan et al. found a cross-shift decline in pulmonary function [forced expiratory volume in one second (FEV1)] associated with increased exposure to levoglucosan, a byproduct of biomass burning measured in PM (< 10 μm) (22). Similarly, among firefighters responding to a controlled burn, Slaughter et al. found a measured decline in pre- and post-shift FEV1; however, the decline was not significantly associated with accompanying exposure to PM (< 3.5 μm) (23). And in non-occupational settings, emerging evidence established an association between acute respiratory effects, including pulmonary function declines and reduced exercise performance, and short-term exposure to diesel exhaust and PM (< 1 and <2.5 μm) in healthy individuals and in those with asthma, chronic obstructive pulmonary disease, and ischemic heart disease (24–29). Based on this literature, we expect that e-waste workers exposed to high concentrations of PM and co-occurring inhalation hazards [e.g., carbon monoxide (CO), PAHs, metals] are likely to exhibit accelerated declines in pulmonary function. The severity of declines may differ by specific work activities.

Using data collected at the Agbogbloshie informal e-waste recovery site and a reference community in Accra, Ghana, this study evaluates whether acute changes in pulmonary function are associated with personal PM using a highly sensitive cross-shift study design. Cross-shift studies enable each study participant to serve as their own referent, reducing the impact of confounding, and do not require a long-term follow up. The first aim is to evaluate the association between cross-shift changes in pulmonary function and personal exposures to PM_1_ (< 1 μm), PM_2.5_ (< 2.5 μm), coarse fraction PM (2.5–10 μm), and self-reported pre-shift exposures among e-waste recovery workers and a reference population. The second aim is, among e-waste workers only, to evaluate the association between cross-shift changes in pulmonary function and activities performed during the work shift. The results will contribute to the limited epidemiologic evidence on acute respiratory health effects associated with unusually high personal PM concentrations among e-waste workers in Accra, Ghana.

## 2 Materials and methods

### 2.1 Study sample

Study participants were enrolled in the West Africa-Michigan CHARTER II for GeoHealth (GeoHealth) (*N* = 207), a longitudinal cohort study with four waves of data collection designed to assess environmental and occupational health among e-waste recovery workers at the Agbogbloshie e-waste site in Accra, Ghana. Eligible participants (*N* = 131) included male e-waste recovery workers from the Agbogbloshie e-waste site (*N* = 81) and male residents from a reference population (*N* = 50) living in the Madina Zongo (MZ) district of Accra who completed personal shift sampling during the second (August 2017–October 2017) and/or the third wave (January-April 2018) of data collection.

Details on the geographic setting and participant recruitment at Agbogbloshie have been described previously (18, 19). An attempt to enroll an inception cohort of e-waste workers at Agbogbloshie was unsuccessful, as information on when or if a new worker arrived to the site was unavailable. The MZ community members were selected as an appropriate reference population based on their geographic separation from e-waste associated pollutants and similar religion and region of origin to the e-waste worker population. In this study, the role of the reference population is to provide otherwise unavailable background levels of personal PM inhalation exposure and respiratory health of Accra residents with similar socio-, cultural- and economic characteristics of e-waste workers. MZ is comprised of housing structures and small-scale businesses serving community needs and is surrounded by a high traffic four-lane road (N4). A sufficient number of individuals in both study locations volunteered to participate. Compensation at each wave included 30 Ghana Cedis (~7 USD), lunch and a t-shirt. Informed consent was obtained and all study questionnaires were administered by trained, local interpreters in the preferred language of the participant. Institutional Review Board approval was obtained from the University of Ghana and the University of Michigan.

### 2.2 Data collection

#### 2.2.1 Baseline health interviews

A baseline health survey was completed for each participant at their initial study visit (wave I or wave II). The survey included questions on socio-demographics, tobacco use and indoor cooking habits adapted from the Ghana Demographic Health Survey (30). Standard respiratory symptoms (see Table 1) were derived from the Medical Research Council questionnaire (MRCQ) on respiratory symptoms (31). Symptoms included: usual cough; usual phlegm production; phlegm production longer than 3 months; chronic bronchitis (defined as cough longer than 3 months and phlegm production longer than 3 months); breathlessness when walking; severe breathlessness when walking; wheezing; chest tightness; and shortness of breath.

**Table 1:** Socio-demographics of the GeoHealth cohort with valid cross-shift pulmonary function tests (*N* = 120; 73 E-waste workers and 47 members of a reference population), Accra, Ghana, 2017–2018.

**Characteristic**		**E-waste**	**Reference**	**p-value**
Sex (%)	Male	100	100	NA
Age (years) [mean (SD)]		26.5 (6.6)	30.7 (9.2)	< 0.01
Country of origin (%)	Ghana	100	97.8	0.39
	Other	0	2.2	
Region of origin (%)	Northern	100	33.3	< 0.01
	Other	0	44.4	
	Accra	0	22.2	
Daily income^a^ (%)	< = GHS 20	15.1	18.6	0.23
	GHS 21–60	65.8	53.5	
	GHS 61–200	13.7	11.6	
	>200 GHS	5.5	16.3	
Religion (%)	No religion	2.7	0	0.010
	Other	2.7	17.0	
	Muslim	94.5	83.0	
Marital status (%)	Single	45.2	70.2	0.009
	Married	54.8	29.8	
Education (%)	No education	27.4	19.1	0.010
	Less than secondary	60.3	44.7	
	Secondary	12.3	29.8	
	Higher	0	6.4	
Home type (%)	Rented room	27.4	40.4	< 0.01
	Rented/owned Kiosk	43.8	6.4	
	Outdoors/ mosque	1.4	0	
	Own home	27.4	53.2	
Use of indoor cooking (%)	Yes	16.4	51.1	< 0.01
	No	83.6	48.9	
Method of cooking (%)	Open fire	0	2.2	< 0.01
	Stove/Coal pot WITH vent	5.5	8.7	
	Stove/Coal pot WITHOUT vent	0	4.3	
	LPG cook stove	2.7	23.9	
	Electricity	6.8	10.9	
	Do not cook indoors	84.9	50.0	
Sleep in same room as cooking (%)	Yes	9.9	17.4	0.36
	No	90.1	82.6	
Tobacco smoke status (%)	Current	25.0	6.5	0.014
	Former	1.4	2.2	
	Never	73.6	91.3	

^a^1 USD was equivalent to ~4.42 GHS at the time of the study.

#### 2.2.2 Personal inhalation exposure to particulate matter

Personal inhalation exposure to size-specific PM was estimated using measurements from a 5-channel optical particle counter (Aerocet 831, Met One Instruments, Oregon, USA) worn in a sampling backpack by each participant during a partial work-shift (e-waste and reference population) or, among reference participants who did not go to work, during completion of daily activities. Specific details on the sampling protocol and how the device works have been described previously (18). The device continuously measures (once every minute) particle counts (sizes <1, <2.5, <4, and <10 μm in aerodynamic diameter) from the participant's breathing zone and converts them into size-specific mass measurements (as μg m^−3^). Measures of PM_10_ exceeding 2000 μg m^−3^ (0.3% of the data, *n* = 369 min) were censored to avoid potential bias from coincidence error (i.e., when multiple small particles appear as one larger particle resulting in an overestimate of large particles). PM_2.5 − 10_ was derived by subtracting PM_2.5_ from PM_10_. Shift averages for PM_1_, PM_2.5_ and PM_2.5 − 10_ were derived for each participant. Shift peak concentrations were defined as the maximum 5-min means for PM_1_, PM_2.5_ or PM_2.5 − 10_ concentrations for each participant.

Deployment of the personal sampling backpacks for both study groups occurred between 8 and 11 AM and retrieval occurred between 12 and 3 PM. Participants were initially asked to wear the sampling backpacks for a minimum of 6 h. The sampling time was initially reduced to 4 h during wave 2 after learning that the majority of workers stopped working after 4 h; reducing the sampling period limited potential confounding from PM exposure caused by the performance of non-e-waste related activities while having the added benefit of reducing participant burden. A subsequent reduction in sampling duration from 4 to 2 h occurred during the Harmattan season (wave 3) when winds from the Saharan Desert transported sand and dust across the region between November and February as the high PM levels could compromise measurements obtained by some of the equipment in the sampling backpacks. The Harmattan winds were expected to impact the personal sampling equipment and inhalation exposure concentrations of both the e-waste and reference populations; studies have shown 4-fold increases in PM_2.5_ concentrations across the Greater Accra Metropolis during Harmattan season in comparison to non-Harmattan seasons (32).

#### 2.2.3 E-waste recovery activities

Image-derived time-activity data were generated for a sub-cohort of e-waste worker participants (*n* = 50) during their work-shifts. Time-lapse images (one per minute) were taken using a wide-angle GoPro Hero4© camera mounted to the shoulder strap of the personal sampling backpack. Details on how the images were processed to derive time-activity data have been described previously (18). Activity categories included: burning e-waste, dismantling e-waste, sorting/loading e-waste, buying/selling e-waste, transporting e-waste and scrap materials, other e-waste activities, other work activities unrelated to e-waste recovery, use of a motorcycle or car, walking, bicycling, smoking or in the presence of tobacco smoke, and not actively working (i.e., sitting, eating or drinking, cell phone use, prayer, and communicating with others).

#### 2.2.4 Pre-and post-shift interviews and self-reported pre-shift exposures

Pre-shift and post-shift interviews were performed for each participant prior to the deployment of the personal sampling backpack (between 8 and 11 AM) and after backpack retrieval (between 12 and 3 PM). The pre-shift survey included questions on current respiratory symptoms and pre-shift exposures. Current respiratory symptoms included: irritation or burning of the eyes, nose or throat; cough; wheezing or whistling sound in chest; shortness of breath, difficulty catching your breath, or a smothering feeling; and chest tightness. Pre-shift exposures include working “yesterday” (the day before the pulmonary function test) and working prior to the pre-shift pulmonary function test (same day). The post-shift survey included the same questions on current respiratory symptoms and tobacco use during the shift. Incident respiratory symptoms were defined as those reported on the post-shift questionnaire and not on the pre-shift questionnaire.

#### 2.2.5 Cross-shift pulmonary function

Pre-shift and post-shift pulmonary function tests were performed at the same time as the pre- and post-shift interviews, i.e., prior to the deployment of the personal sampling backpack (between 8AM and 11AM) and after backpack retrieval (between 12PM and 3PM). Pulmonary function was assessed using the handheld EasyOne Diagnositic spirometry device (NDD Medical Technologies, Andover, MA) following the guidelines of the American Thoracic Society (ATS) (33). Two examiners, a local physician and emergency medical technician, were trained on how to use the device and administer the test. Before beginning the test, age, height and weight were recorded and the maneuver was demonstrated. Participants were coached to take a maximal inspiration and then blast the air out of their lungs into the device as hard, fast and as long (minimum 6 s) as they could. Participants performed a maximum of six maneuvers, and were asked to stop after performing three maneuvers that were considered adequate by both the examiner and an automated quality grade. The device stored the best three maneuvers for each participant.

Pulmonary function parameters of interest included cross-shift changes in forced vital capacity (FVC), forced expiratory volume in one second (FEV1) and the FEV1/FVC ratio. Before calculating cross-shift measures, two trained reviewers graded the acceptability of each of these parameters for each maneuver following the acceptability criteria of the ATS (33). The duration of the exhalation had to be >4 seconds with a plateau on the volume-time curve showing no change in volume for at least 1 s. A third reviewer was consulted in the event of a discrepancy. As per ATS criteria, the best FEV1 and best FVC values were used even if from two different maneuvers and, when possible, the FEV1/FVC ratio was calculated from the curve with the largest sum FEV1 plus FVC. FEV1 and FVC values were graded for between-maneuver repeatability criteria (i.e., a difference -< 0.15 L between the two largest of each values). However, repeatability was not a basis for exclusion as this has been shown in prior research to produce selection bias (34). “Valid” measures are those that met acceptability criteria, and “reproducible” measures are valid measures that also met repeatability criteria. Cross-shift change in FEV1, FVC and FEV1/FVC ratio were calculated for all participants with valid paired pre- and post-shift FEV1, FVC or FEV1/FVC ratio measures. A cross-shift change is defined as the percent change in FEV1, FVC and FEV1/FVC ratio per hour and calculated as: [(post-shift value – pre shift value) / pre-shift value ^*^ 100] / shift length (hours)].

Valid test results were expressed as the percentage of the predicted values expected for a “normal” population of the same sex, age, height and race using equations derived from a population-based study of 7, 429 asymptomatic, non-smoking participants of the National Health and Nutrition Examination Survey (NHANES)-III (35). While no established predicted values exist for the African continent or Ghanaians in particular (36), African-Americans are expected to share substantial common ancestry. The NHANES-III sample used to create the predicted values includes 2, 508 African-American participants out of a total of 7, 429 (35).

### 2.3 Statistical methods

Study groups (e-waste and reference population) were compared across socio-demographic characteristics, baseline respiratory health status, baseline pulmonary function (absolute values and percent predicted), personal inhalational exposure to PM_1_, PM_2.5_, PM_2.5 − 10_, pre-shift exposures and incident respiratory symptoms. The primary health outcomes included cross-shift changes in pulmonary function measures (FEV1, FVC, and FEV1/FVC ratio). The distributions of PM measures were log-normal. A binary log transformation was used; a one-unit change in PM represented a two-fold or doubling effect of PM exposure on the outcome. Associations between cross-shift changes and exposures were estimated using linear mixed effects (LME) models. LME models include a random intercept for participant to account for correlated outcomes among participants in more than one wave of the study. In cases when the random effect for subject was ~0, linear regression was used to avoid overfitting the model. The main effects for study group, PM_1_, PM_2.5_ and PM_2.5 − 10_ (shift mean and peak concentrations), and pre-shift exposures are presented for each of the primary health outcomes. All models were adjusted for *a priori* confounders including age, height, the use of cigarettes during the shift, study wave, and day of week. Participants with a history of asthma (*n* = 2) were excluded from the regression analyses. No participants reported a history of tuberculosis. Differences in the effects of personal PM and pre-shift exposures on cross-shift change in pulmonary function between study groups were tested using both interaction terms added to the fully adjusted models and stratified models. Among e-waste workers only, linear regression models adjusted for age, height and smoking cigarettes during the shift were used to estimate the associations between activities performed during the shift and cross-shift changes in pulmonary function. Activity was parameterized as both a binary variable (performed the activity or not) and a count variable (number of minutes spent performing the activity). All analyses were accomplished using the statistical software R (37).

## 3 Results

### 3.1 Sample

Personal sampling was conducted during 175 monitored shifts (from 131 unique participants; 81 e-waste workers and 50 members of the reference population). Complete data sets (including personal PM and a valid cross-shift FEV1 and/or FVC) were available for 156 shifts (120 unique participants; 73 e-waste workers and 47 members of the reference population). More e-waste worker participants (9%) than reference population participants (6%) were removed from the analysis due to the lack of a cross-shift pulmonary function measures that met ATS acceptability criteria. Socio-demographic characteristics of participants with complete data did not differ significantly from the full cohort (data not shown).

Unexpectedly, the population of MZ residents chosen as the reference population for this study differed from the e-waste worker population across the majority of socio-demographic characteristics (Table 1). In comparison to the reference population, e-waste workers were younger, with lower incomes and education, had a higher prevalence of current cigarette smokers (25% vs. 6%) and lived less frequently in an abode where indoor cooking routinely took place (16% vs. 51%). Among the reference population that did cook indoors, liquid petroleum (23.9%) or electric stoves (10.9%) were most common followed by (non-electric) stove or coal pots with (8.7%) and without vents (4.3%). The majority of both populations were Muslim in a majority Christian city and country. Among the 70% of those currently employed in the reference population, “current” jobs included traders (*n* = 15), skilled workers (e.g., tailors, electricians) (*n* =14), *tro-tro* (public van) drivers and driver assistants (*n* = 7) and other (*n* = 9).

### 3.2 Baseline respiratory health status

There were no self-reported cases of tuberculosis and only two cases of asthma confirmed by a doctor among the sub-cohort (Table 2). E-waste workers reported a higher prevalence of all of the 10 respiratory symptoms queried in comparison to the reference population, but only wheezing (23.6% vs. 8.5%, *p*-value: 0.049) met statistical significance at the 0.05 alpha level.

**Table 2:** Self-reported respiratory health by study groups among the GeoHealth cohort (*N* = 120; 73 E-waste workers and 47 members of a reference population), Accra, Ghana, 2017–2018.

**Self-reported respiratory health**	**E-waste**	**Reference**	**p-value**
Age (years) [mean (SD)]	26.5 (6.6)	30.7 (9.2)	< 0.01
Height (cm) [mean (SD)]	171.4 (6.7)	173.8 (7.2)	0.06
Weight (kg) [mean (SD)]	70.9 (9.7)	73.2 (13.2)	0.28
Body mass index [mean (SD)]	24.1 (2.6)	24.2 (3.7)	0.91
Asthma, ever (%)	1.4	2.2	1.00
TB, confirmed by doctor (%)	0	0	NA
Usual Cough (%)	30.1	21.3	0.39
Cough, longer than 3 months (%)	13.7	12.8	1.00
Usual phlegm production (%)	25	21.3	0.67
Phlegm production, longer than 3 months (%)	13.7	10.6	0.83
Chronic bronchitis (%)	6.8	4.3	0.70
Breathlessness when walking (%)	8.3	2.1	0.24
Severe breathlessness when walking (%)	6.9	2.1	0.40
Wheezing (%)	23.6	8.5	0.049
Chest tightness (%)	32.9	17.0	0.089
Shortness of Breath (%)	15.1	6.4	0.24

### 3.3 Exposure to particulate matter

The average duration of monitored shifts was longer for the reference population (265.9, range 171–399 min) than the e-waste workers (230.3, range: 148–370 min); 18% of e-waste workers had a shift length < 3 h in comparison to 3% of the reference population. Mean and peak personal PM_1_, PM_2.5_ and PM_2.5 − 10_ concentrations for each participant's shift are summarized by study group in Table 3. Mean and peak personal PM_1_, PM_2.5_ and PM_2.5 − 10_ concentrations were significantly higher (*p* < 0.001) among the e-waste workers in comparison to the reference population (Table 3). The prevalence of tobacco use during the work shift was also higher among e-waste workers than the reference population (86% vs. 14%, *p* < 0.001). A significantly larger proportion of e-waste workers in comparison to the reference population reported working the day before (69% vs. 31%, *p* < 0.001) and working prior to the pre-shift pulmonary function test (74% vs. 26%, *p* < 0.001) (Table 3). Less than half of the reference participants (39%) reported working *during* the shift; among those that did, work activities associated with the highest tertiles of PM_1_, PM_2.5_ and PM_2.5 − 10_ concentrations included selling marijuana, “trading,” “digging” and “tiling.”

**Table 3:** Measured and self-reported exposures during the work-shift and prior to the pre-shift pulmonary function test (PFT) by study group, *N* = 120 unique participants and *N* = 156 work-shifts in the GeoHealth cohort, Accra, Ghana, 2017–2018.

		**Total**	**E-waste**	**Reference**	***p*-value**
N work-shifts		156	92	64	
**Personal inhalation exposure (**μ**g m**^−3^**)**					
PM1, shift mean	Median (IQR)	38.2 (33.8)	51.4 (32.2)	26.3 (12.8)	< 0.001
	Mean (SD)	46.2 (27.3)	57.6 (26.4)	29.6 (18.6)	< 0.001
PM_1_, shift peak	Median (IQR)	104.4 (103.7)	136.7 (102.0)	54.5 (52.8)	< 0.001
	Mean (SD)	123.9 (86.0)	156.1 (84.6)	76.5 (63.3)	< 0.001
PM_2.5_, shift mean	Median (IQR)	51.3 (37.7)	63.8 (28.9)	32.9 (10.7)	< 0.001
	Mean (SD)	55.8 (27.7)	69.6 (24.8)	35.6 (17.4)	< 0.001
PM_2.5_, shift peak	Median (IQR)	142.1 (144.6)	173.0 (111.3)	68.5 (83.5)	< 0.001
	Mean (SD)	159.5 (102.4)	195.7 (93.9)	106.6 (91.2)	< 0.001
PM_2.5 − 10_, shift mean	Median (IQR)	66.4 (51.7)	77.4 (65.5)	47.4 (34.9)	< 0.001
	Mean (SD)	86.6 (75.4)	101.1 (76.9)	65.3 (68.4)	< 0.001
PM_2.5 − 10_, shift peak	Median (IQR)	221.3 (254.8)	257.5 (256.6)	170.4 (199.9)	0.004
	Mean (SD)	314.1 (283.8)	331.6 (249.0)	288.5 (328.8)	0.004
Smoked cigarettes during the shift, self-report	Yes	28	24 (85.7)	4 (14.3)	0.001
**Pre-shift exposures**					
Worked “yesterday” (day prior to PFT)	Yes	113	78 (69.0)	35 (31.0)	< 0.001
Worked prior to pre-shift PFT (same day)	Yes	73	54 (74.0)	19 (26.0)	0.001

### 3.4 Cross-shift change in pulmonary function

Among the 156 monitored shifts with complete data, a total of 153, 123 and 120 cross-shift measures of FEV1, FVC and FEV1/FVC ratio were obtained. Of the 156 eligible sessions, 46% (*n* = 72) were reproducible (i.e., a difference <0.15 L between the two largest of each values) (33) (Supplementary Table 1). The proportion of reproducible pulmonary function maneuvers among e-waste (56%) and reference population (54%) participants were similar.

Pre-shift (baseline) pulmonary function measures for the total cohort and by study group are described in Table 4. Average pre-shift FEV1 and FVC measures for the whole cohort were 86% and 90% of the predicted value for a normal population of the same age, height and race, respectively (Table 4). When comparing study groups, pre-shift FEV1 and FEV1/FVC ratio were lower among e-waste workers (Table 4). Pre-shift FVC averages and percent predicted values were modestly higher among e-waste workers in comparison to the reference population. These results were replicated when using only reproducible values (Supplementary Table 1).

**Table 4:** Cross-shift pulmonary function (PF) by study group among the GeoHealth cohort (*N* = 120 unique participants), Accra, Ghana 2017–2018.

		**Overall^a^**	**E-waste**	**Reference**	***p*-value^b^**
	N matched pre- and post-shift PF tests	156	92	64	
	Age (years) [mean (SD)]	28.5 (7.9)	26.7 (6.4)	31.0 (9.1)	0.01
	Height (cm) [mean (SD)]	171.6 (7.0)	170.7 (6.9)	172.9 (7.0)	0.05
	Weight (kg) [mean (SD)]	71.1 (11.3)	70.3 (10.1)	72.4 (12.8)	0.25
Pre-shift PF	FEV1, pre-shift [mean (SD)]	3.1 (0.5)	3.0 (0.5)	3.2 (0.5)	0.06
	FEV1 % predicted [mean (SD)]	86.3 (12.3)	84.9 (13.5)	88.4 (10.2)	0.08
	FEV1 % predicted < 70 = yes (%)	12 (7.8)	9 (9.9)	3 (4.8)	0.39
	Best FVC, pre-shift [mean (SD)]	3.8 (0.6)	3.8 (0.5)	3.7 (0.6)	0.77
	FVC % predicted [mean (SD)]	90.2 (12.9)	91.6 (13.4)	88.3 (11.9)	0.15
	FVC % predicted < 70 = 1 (%)	7 (5.4)	3 (3.9)	4 (7.5)	0.62
	FEV1/FVC Ratio [mean (SD)]	0.8 (0.1)	0.8 (0.1)	0.8 (0.1)	0.01
	Ratio < 0.7 = yes (%)	8 (6.3)	8 (10.7)	0 (0.0)	0.04
Post-shift PF	FEV1, post-shift [mean (SD)]	3.0 (0.5)	2.9 (0.5)	3.1 (0.5)	0.04
	FEV1 % predicted [mean (SD)]	84.5 (13.1)	82.8 (14.2)	86.9 (10.9)	0.06
	FEV1 % predicted < 70 = 1 (%)	22 (14.2)	17 (18.7)	5 (7.8)	0.09
	Best FVC, post-shift [mean (SD)]	3.7 (0.6)	3.7 (0.6)	3.7 (0.6)	0.83
	FVC % predicted [mean (SD)]	88.2 (13.6)	89.1 (15.4)	86.8 (10.4)	0.34
	FVC % predicted < 70 = yes (%)	8 (5.8)	5 (6.1)	3 (5.5)	1.00
	FEV1/FVC Ratio [mean (SD)]	0.8 (0.1)	0.8 (0.1)	0.8 (0.1)	0.01
	Ratio < 0.7 = yes (%)	10 (7.4)	10 (12.3)	0 (0.0)	0.02
Cross-shift change	% Change in FEV1 [mean (SD)]	−1.9 (8.2)	−2.2 (9.4)	−1.5 (6.4)	0.61
	% Change in FVC [mean (SD)]	−1.0 (6.7)	−1.2 (7.1)	−0.8 (6.0)	0.77
	% Change in FEV1/FVC ratio [mean (SD)]	−0.5 (4.7)	−0.8 (5.2)	−0.1 (3.9)	0.41

^a^Number of matched sessions with a valid pre and post-shift FEV1, FVC and FEV1/FVC ratio were 153, 123 and 120, respectively; ^b^T-test p-values are comparing exposed and reference populations.

Unadjusted cross-shift changes in FEV1 and FVC were negative for both study groups, indicating a decrease in pulmonary function throughout the work-shift (Table 4). Cross-shift changes in FEV1/FVC ratios were not observed in either study group. E-waste workers had larger cross-shift changes in FEV1 (−2.2 + 9.4%) and FVC (−1.2 + 7.1%) than the reference population (−1.5 + 6.4% and −0.8 + 6.0% respectively); however, the differences did not reach statistical significance. More e-waste workers (18.7%) than the reference population (7.8%) had a post-shift FEV1 percent predicted below 70% (*p*-value: 0.094). When using only reproducible results, the reference population had greater decreases in both FEV1 and FVC, however, the differences between groups did not reach statistical significance (Supplementary Table 1).

### 3.5 Association between PM and cross-shift change in pulmonary function

**Figure 1:**
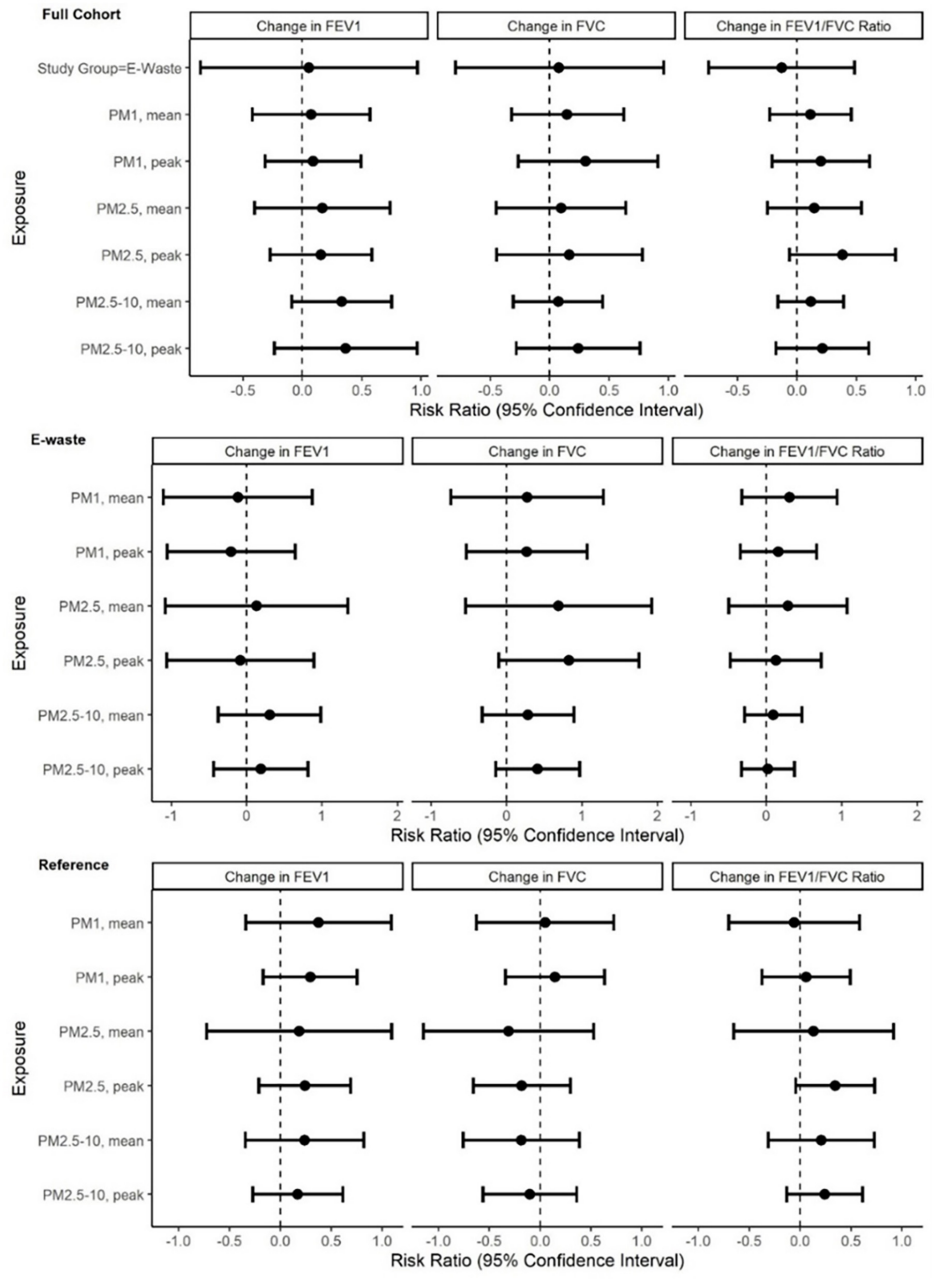
Associations between percent change in pulmonary function outcome per doubling of personal inhalation exposure to PM_1_, PM_2.5_ and PM_2.5 − 10_ for the full cohort (*N* = 156) and stratified by study group (*n* = 92 E-waste; *n* = 64 Reference participants) in the GeoHealth cohort at Agbogbloshie, Accra, Ghana, 2017–2018. Effect estimates and 95% confidence intervals were derived using linear mixed effects models with a random intercept for participant to account for correlated outcomes among participants in more than one wave of the study. Models were adjusted for age, height, the use of cigarettes during the shift, study wave, and day of week.

Measures of association between personal inhalation exposure to PM_1_, PM_2.5_ or PM_2.5 − 10_ and percent change per hour in FEV1, FVC, and FEV1/FVC ratio adjusted for age, height, the use of cigarettes during the shift, study wave, and day of week for the full sample and stratified by study group are summarized in Figure 1 and Supplementary Table 2. As a whole, the results show no signal that increasing levels of PM were associated with a decrement in pulmonary function throughout the work-shift in either study population. Contrary to our expectations, the directions of the estimated risk ratios were overwhelmingly positive; however, the 95% confidence intervals for all tested associations crossed zero, indicating no effect of PM on pulmonary function. When using only reproducible pulmonary function values, we still did not see any negative associations between PM exposure concentrations and cross-shift change in PF. We did, however, observe a positive association between a doubling of mean and peak PM_1_ concentrations throughout the work-shift with FEV1 and FVC in the full cohort after adjusting for age, height and smoking during the shift (Supplementary Figure 1).

### 3.6 Association between pre-shift exposures and cross-shift change in pulmonary function

Working “yesterday” was associated with a 1.22% decrease in FVC per hour (95% CI: −2.18, −0.27) after adjusting for age, height, use of cigarettes during the shift, wave of data collection and day of week (Supplementary Table 2). When stratified by study group, e-waste worker participants who reported working “yesterday” had an average 2.4% (95% CI: −4.04, −0.81) and 1.2% (95% CI: −3.07, 0.69) cross-shift decrement in FVC and FEV1, respectively, while essentially no association was observed among the reference population (Supplementary Figure 2, Supplementary Table 3). When using only reproducible values, the same trend was observed (Supplementary Figure 3). Working prior to the pre-shift pulmonary function test was not associated with a measured increase or decrease in FEV1, FVC or the ratio in either study group.

### 3.7 Incident symptoms and pulmonary function

E-waste workers had a higher incidence of all symptoms in comparison to the reference population (Supplementary Table 4). More than twice as many e-waste workers than reference population reported incident chest tightness (9.8% vs. 3.1%) cough (17.4% vs. 11.1%), shortness of breath (9.8% vs. 6.2%) and wheezing (16.3% vs. 7.8%). However, the total numbers were small and the difference did not reach statistical significance at the 0.05 alpha level. No statistical associations were observed between incident symptoms and cross-shift changes in pulmonary function (data not shown).

### 3.8 Activities performed by e-waste workers and pulmonary function

The average length and range of time for each activity performed by the e-waste worker participants with image-derived data (*n* = 50) is summarized in Table 5. The most common activities performed among the e-waste workers included not actively working (e.g., sitting, cell phone use, communicating), walking, motorcycle or car use, sorting/ loading and dismantling e-waste. Very few participants (*n* = 5) performed burning e-waste during the monitored work shift.

**Table 5:** Descriptive statistics on image-derived activities performed by e-waste worker participants during their monitored work shift at Agbogbloshie, Accra, Ghana, 2017–2018.

**Activity**	**Mean duration in minutes (range)**	**Number of participants who performed the activity**
Burning e-waste	69.6 (7, 147)	5
Dismantling e-waste	78.4 (3, 238)	8
Buying, selling e-waste	25.5 (16, 35)	2
Transporting materials	28.0 (4, 88)	8
Sorting, loading e-waste	59.7 (5, 170)	10
Motorcycle or car use	32.6 (4, 129)	17
Bicycling	29.5 (17, 37)	4
Walking	31.4 (5, 113)	45
Not actively working	105.2 (9, 225)	43
Presence of tobacco smoke	48.0 (23, 67)	3
Other, e-waste related work	69.7 (16, 116)	3
Other, non-e-waste related work	6.0 (4, 8)	2

**Figure 2:**
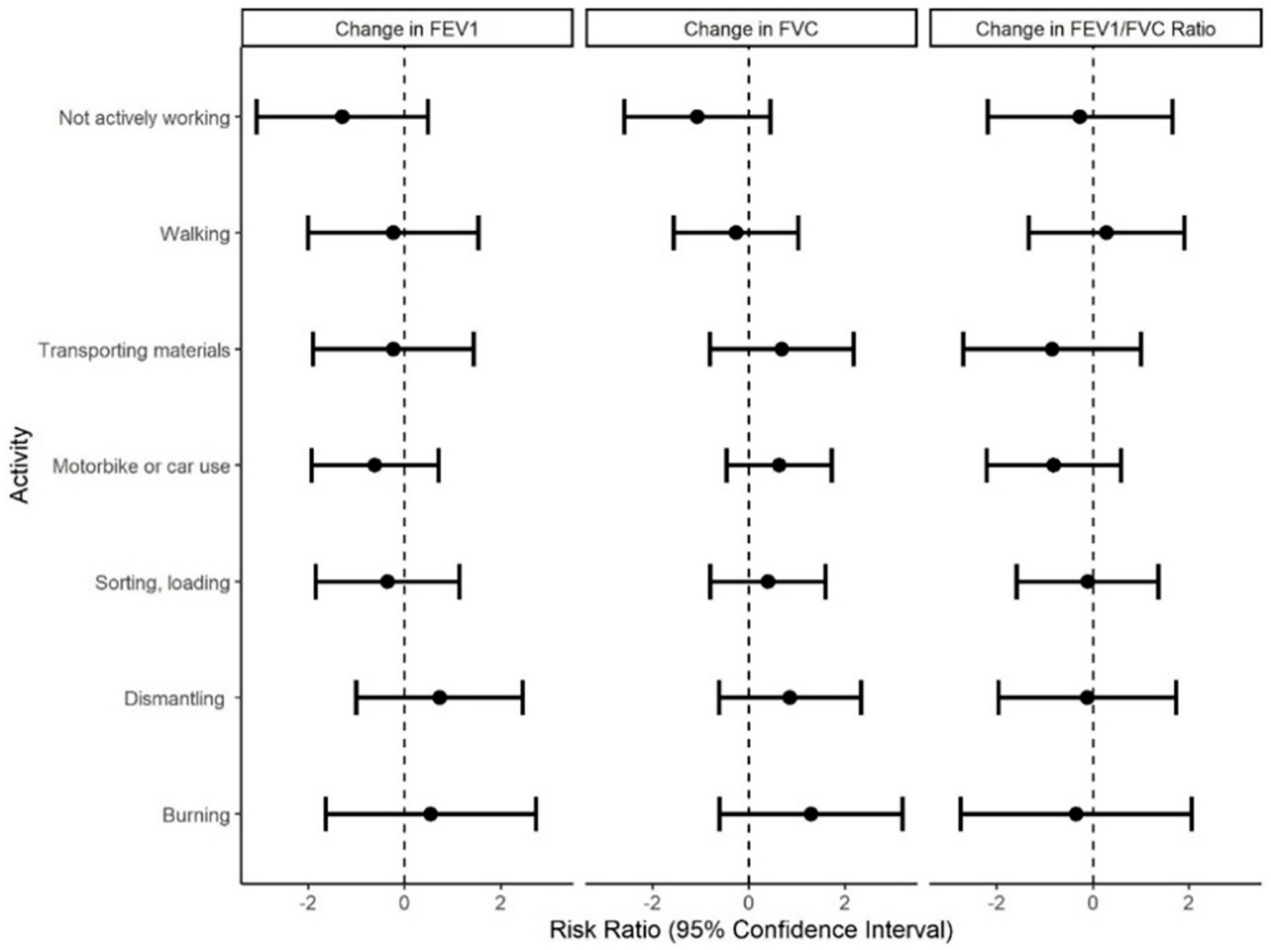
Associations between percent change in pulmonary function outcomes and performance of activities among e-waste recovery workers (*n* = 50) in the GeoHealth cohort at Agbogbloshie, Accra, Ghana, 2017–2018. Effect estimates and 95% confidence intervals were derived using linear regression adjusted for age, height and smoking cigarettes during the shift.

Among the activities performed by five or more participants, comparisons between unadjusted cross-shift changes in FEV1, FVC and FEV1/FVC ratio for those who performed the activity to those who did not showed no measurable differences (data not shown). In models adjusted for age, height and smoking during the shift, associations between all of the activities and percent changes in FEV1, FVC or FEV1/FVC were all close to zero (Figure 2). A modestly protective effect of burning e-waste and dismantling e-waste was found; workers who burned and dismantled e-waste at least once during the work-shift had a 0.38 (−1.84, 2.59) and a 0.69 (−1.11, 2.50) percent increase in FEV1 per hour, respectively, in comparison to those who did not perform those activities at all. Interestingly, not actively working was associated with a −1.05 (−2.88, 0.78) percent decrease in FEV1 per hour in comparison to those who did not perform that activity (i.e., were not ever “not actively working”) at all. When activity was parameterized as a count variable (length of time performing the activity), similar results were found with one exception: an increase in the number of minutes a worker performed dismantling was associated with an improvement in FVC per hour (Supplementary Figure 4).

## 4 Discussion

The aim of this study was to examine the effect of personal inhalation exposure to PM on pulmonary function among e-waste recovery workers and a reference population in Accra, Ghana using a cross-shift study design. Personal inhalation exposure to PM_1_, PM_2.5_ and PM_2.5 − 10_ among e-waste workers at the Agbogbloshie e-waste site were nearly double the levels experienced by the comparison group, Accra residents who live and work near a heavily trafficked road. Both populations, however, experienced mean concentrations of personal PM_2.5_ that far exceeded the WHO's ambient Air Quality Guideline recommendations (24-h mean: 15 μg m^−3^) (38). Although the mean PM concentrations for the e-waste workers in this study would fall below the general particulate occupational standard used by U.S. Department of Labor Occupational Safety and Health Administration (8-h time-weighted average: 5000 μg m^−3^), it is not protective in an informal setting given the likelihood of toxic metals and organic compounds in the PM (20, 39), and because there is nothing approaching the hierarchy of controls at the worksite that is intended to reduce exposure to as low as practicable when toxic substances are present (40). Following an average monitored shift of 4 hours (+44 min), declines in pulmonary function (FEV1 and FVC) were observed among e-waste workers and the reference population. Although declines in FEV1 and FVC were largest among e-waste workers, they did differ significantly from those observed among the reference population. Exposure to personal PM_1_, PM_2.5_ and PM_2.5 − 10_ concentrations was not associated with cross-shift declines in pulmonary function in either study group. Cross-shift changes in pulmonary function were also not significantly associated with e-waste recovery activities performed during the shift.

The lack of a significant association between cross-shift pulmonary function and PM among e-waste workers may be attributable to health-related job selection or the “healthy worker” effect (41–43). Current workers selected for the study may still be able to tolerate high levels of PM exposure and be more resistant to short-term respiratory effects than the workers who have left the job due to health complications (44). In previous studies, E-waste recovery workers have reported leaving their jobs to return to their family homes in other regions of Ghana when health complications arise (45).

There are, however, alternative explanations to our study findings. First, E-waste workers may have experienced a decline in pulmonary function associated with e-waste-related exposures, but only during their initial months of employment and prior to our study. Several studies have observed differential effects of metal working fluids and respirable dust among machinists and coal miners, respectively, according to years of employment; the dose-response curve flattens out with increasing years of employment suggesting a threshold effect (46–48). In our sample, the average length of employment at Agbogbloshie among e-waste participants (8.8 + 6.6 years) was too long to measure initial reductions in pulmonary function (48, 49). Second, we may not have captured a true pre-shift measure of pulmonary function, due to the fact that most e-waste workers reported living at the e-waste site (89%) and having already worked prior to the pre-shift test (74%). Lastly, any cross-shift changes may have been diluted by the diurnal variation in pulmonary function. A limitation of using pulmonary function measures without a resting control is that we do not know how much of the variation is due to natural diurnal variation (49). Given the lack of a true pre-shift pulmonary function assessment and the diurnal variation in pulmonary function, a shift duration of 4 h (+44 min) from morning to mid-afternoon may have been too short to capture a measurable change in lung function due to occupational exposures.

The steeper decline in pulmonary function among e-waste workers who reported working the day before the cross-shift pulmonary function assessments, in comparison to those who did not, provides some evidence of respiratory health effects from e-waste associated PM pollution occurring in the range of 24–96 h before the measurements took place. Time-series studies using distributed lag models to examine the association between ambient air pollution and health outcomes, such as mortality, asthma and hospitalizations for myocardial infarction, have found larger effect sizes for 1 to 6 day lag exposures in comparison to same-day exposure (50–53).

The high incidence of respiratory symptoms, particularly cough (17%) and wheeze (16%), reported by e-waste workers following an average of 4 h of work is notable. Prior studies have observed a high prevalence of self-reported respiratory symptoms among e-waste workers (45) and children living in the vicinity of an e-waste site (15). Together, these findings provide evidence of an acute respiratory response to e-waste associated pollutants that was undetected by spirometry in our study. In research among World Trade Center responders exposed to toxic dust from the collapse of the World Trade Center in 2001, the frequency and severity of respiratory symptoms were found to be associated with small airways abnormalities that were initially undetected using spirometry (54, 55). In other words, the use of routine spirometry may not have been sensitive enough to detect abnormalities in lung function among symptomatic e-waste workers.

Given the high variability in pulmonary function outcomes, spirometry, as performed in this study, may have had low levels of accuracy. The standard deviations in valid cross-shift FEV1 and FVC response variables for the whole cohort, +8.25 and +6.67, respectively, were high. In a sensitivity analysis, we compared the residual variance for cross-shift FEV1 and FVC response variables between study groups using a distributional model adjusted for age, height and the use of cigarette smoking during the shift (“brms” package). We found that the residual variance was significantly higher for FEV1 (95% CI: 2.33, 3.14) and FVC (95% CI: 1.88, 2.60) among the e-waste study group in comparison to the reference population. Unequal variance may be a result of field conditions, unmeasured factors, or challenges in eliciting a valid pulmonary function test among e-waste workers. There is some evidence that repeated pulmonary function maneuvers exacerbate airflow narrowing among individuals with prevalent obstructive lung diseases, including asthma, making it harder to achieve valid test results (56). When using only reproducible pulmonary function values, the main results did not change; however, the reproducible models had limited power and may be excluding participants with accelerated declines in pulmonary function rather than those with measurement error (34) .

The application of a gold standard, cross-shift study design, in an informal occupational setting is a strength of this study. The combination of spirometry to assess pre- and post-shift pulmonary function with continuous measures of personal inhalation exposure to three PM sizes (< 1, < 2.5 and 2.5–10 μm) provided a rich dataset from which causal evidence can be generated, in addition to contributing to the limited available evidence on respiratory health among residents of Accra, Ghana. Continuous PM concentrations allowed us to examine the effects of both daily mean and the very high peak PM concentrations which are unique to e-waste recovery, on pulmonary function. Lessons related to the measurement of cross-shift pulmonary function among informal e-waste workers can inform future studies in other non-traditional occupational settings that are grappling with similar challenges (e.g., the lack of separation between work and life activities).

This work has several limitations. Breathing zone concentrations of PM were estimated using optical measurements rather than gravimetric mass measurements (considered the reference approach), which may be associated with measurement error. Based on simulations and experiments described in our previous work, it was concluded that the optical-based measurements underestimated the true PM concentrations and that particle losses were greatest among the larger sized particles (i.e., PM_2.5 − 10_) (19, 39). Pre-shift pulmonary function measurements were limited by the fact that most e-waste worker participants reported living and sleeping on or near to the worksite. The shift duration may have been too short to capture significant changes in pulmonary function considering the lack of a true “pre-shift” assessment. Limited statistical power, particularly among the reference population, impeded our ability to make comparisons that we would have liked to make (i.e., between e-waste workers who did not work yesterday *and* did not work prior to the pre-shift assessment with the same subcategories among the reference population). The absence of an inception cohort limited our ability to observe possible early decrements in lung function experienced among new workers in comparison to seasoned workers. The high degree of variability in the pulmonary function data may be indicative of inaccuracies; such uncertainty in the data is hard to overcome. The sample size of e-waste worker participants with image-derived activity data was too small to establish reliable results.

Future studies in informal settings where workers commonly live and work in the same vicinity should aim to monitor participants for a longer period, possibly over the course of multiple, consecutive workdays starting with the lightest exposure day. With participant cooperation, performing spirometry at multiple time-intervals could help distinguish between diurnal variation and exposure-related change in pulmonary function. We also recommend the use of a stratified recruitment strategy to include workers with varying lengths of employment and, if possible, a cohort of former e-waste recovery workers. If possible, it would be helpful to perform spirometry or alternative techniques for observing distal airway function in a health center using equipment that allows medical staff to review the results immediately to avoid uncertainty in the data. Alternative longitudinal study designs that estimate total or cumulative exposure and account for exposure mixtures are also needed.

## 5 Conclusions

E-waste recovery is associated with high concentrations of PM pollution (13, 18, 57). The short- and long-term respiratory-related occupational health burden due to e-waste associated PM pollution is unknown, but likely to be substantial. In using a cross-shift study design that combined morning and afternoon pulmonary function assessments with personal monitoring of PM pollution, we contributed to a limited knowledge base on acute respiratory health effects from e-waste recovery work. In this sample, cross-shift declines in pulmonary function were not associated with linked PM_1_, PM_2.5_ and PM_2.5 − 10_ breathing zone concentrations. The limitations we encountered in conducting the study, including the inability to capture a true pre-shift pulmonary function assessment among e-waste workers who sleep at the site, an average shift length of < 4 h, and uncertainty in the pulmonary function data, are plausible explanations for the null findings, which should be interpreted with caution. The challenges encountered in this study highlight how social and economic disparities that underlie the growth of informal economies contribute to occupational hazards themselves (58). In informal sectors, where workers live and work in the same vicinity, ensuring a safe place to sleep goes hand in hand with having a safe place to work.

## Data availability statement

The datasets presented in this article are not readily available because of privacy and ethical restrictions. Requests to access the datasets should be directed to jfobil@ug.edu.gh.

## Ethics statement

This study involving humans was approved by the University of Ghana and the University of Michigan. The studies were conducted in accordance with the local legislation and institutional requirements. The participants provided their written informed consent to participate in this study.

## Author contributions

ZL: Conceptualization, Data curation, Formal analysis, Funding acquisition, Investigation, Methodology, Software, Validation, Visualization, Writing – original draft, Writing – review & editing. MO'N: Conceptualization, Funding acquisition, Methodology, Supervision, Writing – original draft, Writing – review & editing. SB: Writing – original draft, Writing – review & editing, Conceptualization, Data curation, Funding acquisition, Investigation, Methodology, Supervision. BM: Formal analysis, Methodology, Writing – original draft, Writing – review & editing. JF: Conceptualization, Data curation, Funding acquisition, Investigation, Methodology, Project administration, Supervision, Writing – original draft, Writing – review & editing. TR: Conceptualization, Data curation, Formal analysis, Funding acquisition, Investigation, Methodology, Project administration, Resources, Supervision, Validation, Writing – original draft, Writing – review & editing.
