# Supplementary material for: Cross-shift changes in pulmonary function and occupational exposure to particulate matter among e-waste workers in Ghana
Source: Front Public Health. 2024 May 9;12:1368112. doi: 10.3389/fpubh.2024.1368112 (PMC11111984; doi:10.3389/fpubh.2024.1368112)
Supplement: Supplementary file 1 [file Data_Sheet_1.docx]

Supplementary Materials

Cross-Shift Changes in Pulmonary Function and Occupational Exposure to Particulate Matter among E-Waste Workers in Ghana

Zoey Laskaris^1*^, Marie S. O’Neill^2^, Stuart A. Batterman^3^, Bhramar Mukherjee^4^, Julius N. Fobil^5^, and Thomas G. Robins^6^

^1^ Department of Epidemiology, University of Michigan School of Public Health, Ann Arbor, MI, United States of America; laskaris@umich.edu

^2^ Department of Epidemiology and Department of Environmental Health Sciences, University of Michigan School of Public Health, Ann Arbor, MI, United States of America; marieo@umich.edu

^3^ Department of Environmental Health Sciences, University of Michigan School of Public Health, Ann Arbor, MI, United States of America; stuartb@umich.edu

^4^ Department of Biostatistics, University of Michigan School of Public Health, Ann Arbor, MI, United States of America; bhramar@umich.edu

^5^ Department of Biological, Environmental and Occupational Health Sciences, University of Ghana School of Public Health, Accra, Ghana; jfobil@ug.edu.gh

^6^ Department of Environmental Health Sciences, University of Michigan School of Public Health, Ann Arbor, MI, United States of America; stuartb@umich.edu

*** Correspondence:**Zoey Laskaris
[laskaris@umich.edu](mailto:laskaris@umich.edu)

**Supplemental Figure 1:** Associations between percent change in pulmonary function outcome per doubling of personal inhalation exposure to PM_1_, PM_2.5_ and PM_2.5-10_ using reproducible pulmonary function data for the full GeoHealth cohort, Accra, Ghana, 2017-2018.


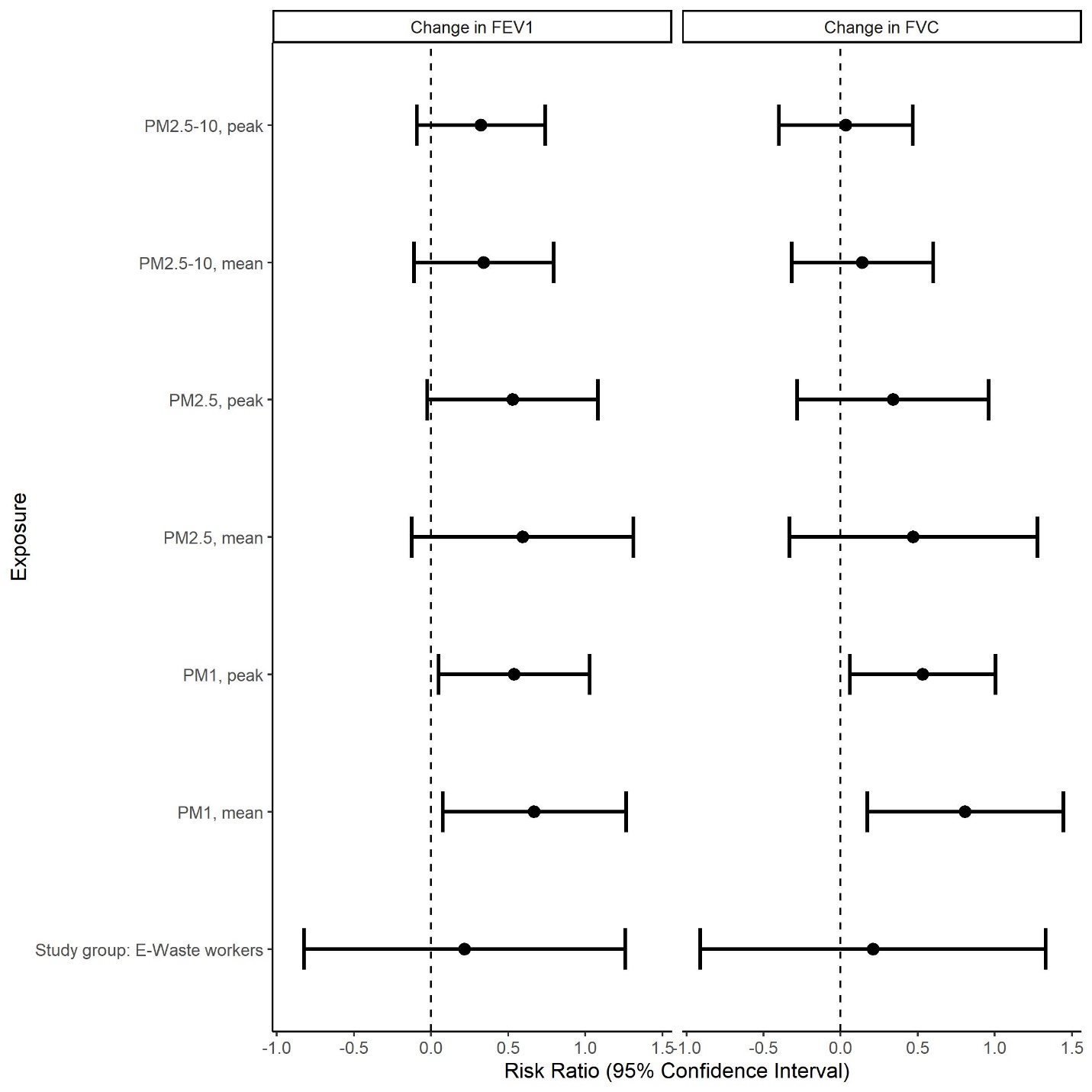


**Supplemental Figure 2:** Effect of working “yesterday” on cross-shift changes in FEV1 (A) and FVC (B) percent predicted values per hour among the GeoHealth cohort, Accra, Ghana, 2017-2018.


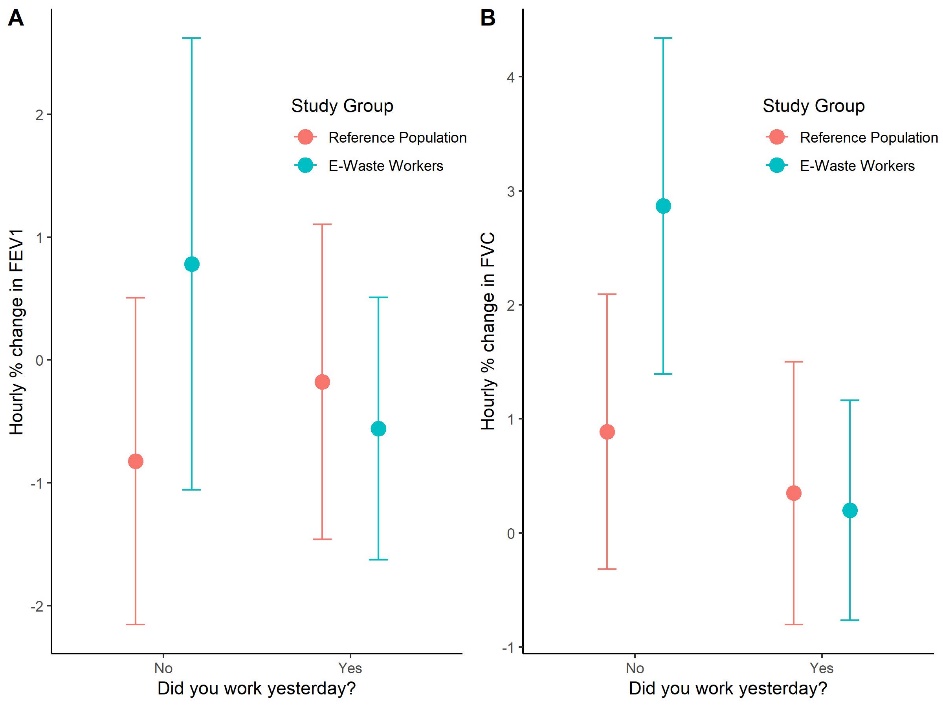


**Supplemental Figure 3:** Associations between percent change in pulmonary function outcome per doubling of personal inhalation exposure to PM_1_, PM_2.5_ and PM_2.5-10_ using reproducible pulmonary function data for the full GeoHealth cohort, Accra, Ghana, 2017-2018.


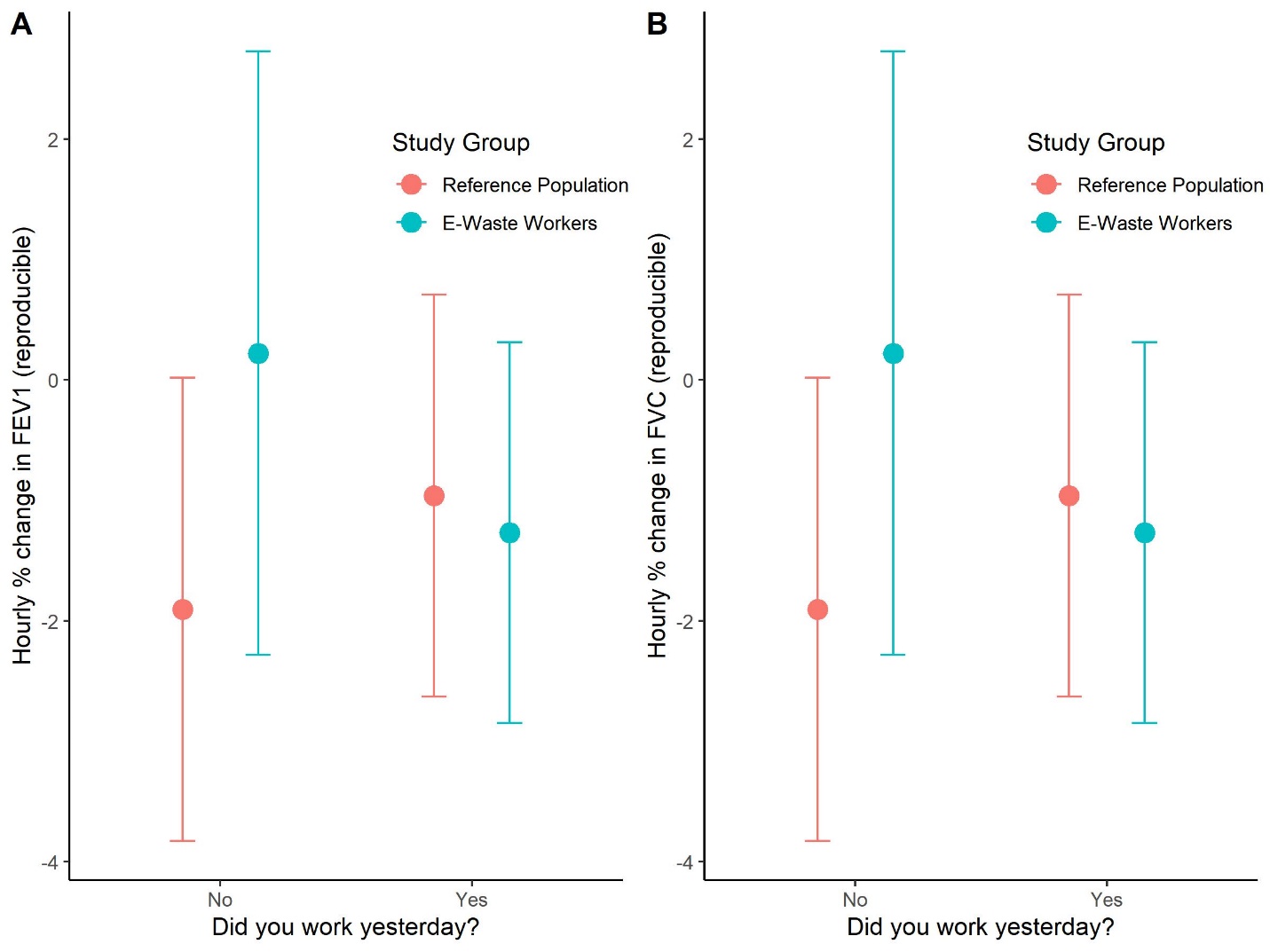


**Supplemental Figure 4**: Associations between e-waste recovery activities (length of time spent performing the activity) and cross-shift changes in pulmonary function among e-waste recovery workers (n=50) enrolled in the GeoHealth cohort at Agbogbloshie, Accra, Ghana, 2017-2018.


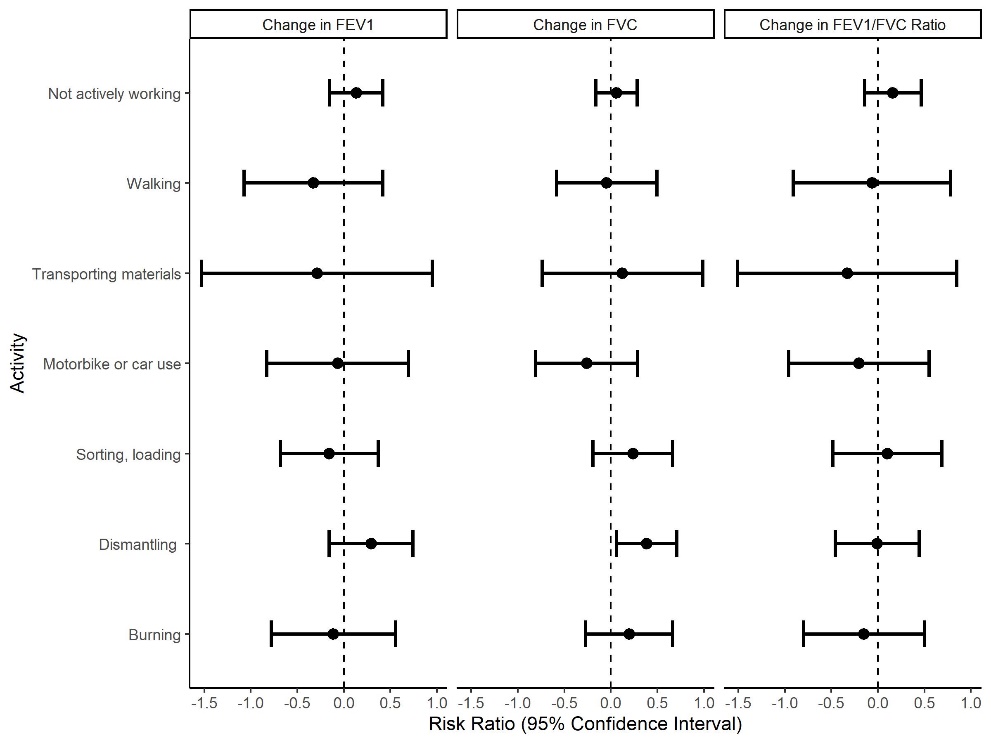


Legend: Linear regression models included a total of 48, 40 and 38 FEV1, FVC and FEV1/FVC ratio values, respectively, and were adjusted for age, height and smoking cigarettes for the duration of the work-shift. A one-unit increase in activity is equivalent to performing the activity for 30-minutes.

**Supplemental Table 1:** Cross-shift pulmonary function by exposure group using reproducible results among the GeoHealth cohort, Accra, Ghana 2017-2018.

|  |  | Overall^a^ | E-waste | Reference | p-value^b^ |
| --- | --- | --- | --- | --- | --- |
|  | N Pulmonary Function Tests | 72 | 44 | 28 |  |
|  | Age (years) (mean (SD)) | 28.56 (7.23) | 29.61 (6.76) | 26.89 (7.75) | 0.12 |
|  | Height (cm) (mean (SD)) | 171.44 (6.80) | 170.57 (5.83) | 172.82 (8.01) | 0.172 |
|  | Weight (kg) (mean (SD)) | 71.65 (11.62) | 71.95 (10.00) | 71.18 (13.97) | 0.784 |
|  | Shift length (mean (SD)) | 247.43 (41.65) | 234.19 (44.93) | 268.23 (24.73) | <0.001 |
| PRE-Shift | FEV1, pre-shift (mean (SD)) | 3.12 (0.47) | 2.98 (0.42) | 3.33 (0.47) | 0.002 |
|  | FEV1 % predicted (mean (SD)) | 87.78 (11.03) | 85.33 (10.92) | 91.46 (10.32) | 0.027 |
|  | FEV1 % predicted <70 = yes (%) | 4 (6.2) | 4 (10.3) | 0 (0.0) | 0.246 |
|  | Best FVC, pre-shift (mean (SD)) | 4.01 (0.59) | 4.02 (0.65) | 3.99 (0.51) | 0.896 |
|  | FVC % predicted (mean (SD)) | 95.54 (12.93) | 97.66 (14.28) | 92.57 (10.52) | 0.25 |
|  | FVC % predicted <70 = 1 (%) | 0 (0.0) | 0 (0.0) | 0 (0.0) | NA |
|  | FEV1/FVC Ratio (mean (SD)) | 0.81 (0.07) | 0.77 (0.06) | 0.85 (0.04) | <0.001 |
|  | Ratio <0.7 = yes (%) | 2 (6.9) | 2 (12.5) | 0 (0.0) | 0.559 |
| POST-SHIFT | FEV1, post-shift (mean (SD)) | 3.06 (0.48) | 2.95 (0.45) | 3.23 (0.47) | 0.017 |
|  | FEV1 % predicted (mean (SD)) | 85.88 (11.95) | 84.22 (12.23) | 88.38 (11.29) | 0.171 |
|  | FEV1 % predicted <70 = 1 (%) | 7 (10.8) | 6 (15.4) | 1 (3.8) | 0.288 |
|  | Best FVC, post-shift (mean (SD)) | 3.95 (0.61) | 3.97 (0.67) | 3.91 (0.52) | 0.747 |
|  | FVC % predicted (mean (SD)) | 93.78 (13.25) | 96.64 (14.52) | 89.78 (10.41) | 0.127 |
|  | FVC % predicted <70 = yes (%) | 0 (0.0) | 0 (0.0) | 0 (0.0) | NA |
|  | Fev1/FVC Ratio (mean (SD)) | 0.80 (0.06) | 0.77 (0.06) | 0.85 (0.04) | <0.001 |
|  | Ratio <0.7 = yes (%) | 2 (6.9) | 2 (12.5) | 0 (0.0) | 0.559 |
| Change | % Change in FEV1 (mean (SD))^2^ | -1.85 (6.31) | -1.10 (6.19) | -2.96 (6.45) | 0.249 |
|  | % Change in FVC (mean (SD))^3^ | -1.52 (5.31) | -1.16 (4.99) | -2.02 (5.87) | 0.639 |
|  | % Change in FEV1/FVC ratio (mean (SD))^4^ | 0.01 (2.29) | 0.41 (2.28) | -0.48 (2.29) | 0.306 |

^a^ Number of matched sessions with a valid pre and post-shift FEV1, FVC and FEV1/FVC ratio were 65, 35, 29, respectively; ^b^ T-test p-values are comparing exposed and reference populations.

**Supplemental Table 2:** Associations between percent change in pulmonary function outcome per doubling of personal inhalation exposure to PM_1_, PM_2.5_ and PM_2.5-10_ for the full GeoHealth cohort, Accra, Ghana 2017-2018.

|  |  |  | ΔFEV1 (% change / hour)^a^ | ΔFVC (% change / hour)^b^ | ΔRatio (% change / hour)^c^ |
| --- | --- | --- | --- | --- | --- |
|  |  | **Model specification** | **RR (95%CI)** | **RR (95%CI)** | **RR (95%CI)** |
| Main effects of exposure group and PM | |  |  |  |  |
|  | **PM_1_, mean** | crude | 0.07 (-0.39 to 0.54, p=0.765) | 0.17 (-0.28 to 0.62, p=0.457) | 0.01 (-0.31 to 0.32, p=0.968) |
|  |  | + covariates^d^ | 0.07 (-0.45 to 0.58, p=0.791) | 0.09 (-0.40 to 0.58, p=0.724) | 0.11 (-0.23 to 0.46, p=0.516) |
|  | **PM_1_, peak** | crude | 0.03 (-0.35 to 0.40, p=0.877) | 0.12 (-0.24 to 0.48, p=0.517) | 0.01 (-0.24 to 0.27, p=0.921) |
|  |  | + covariates | 0.09 (-0.33 to 0.51, p=0.668) | 0.10 (-0.30 to 0.49, p=0.617) | 0.14 (-0.14 to 0.41, p=0.334) |
|  | **PM_2.5_, mean** | crude | -0.00 (-0.53 to 0.53, p=0.999) | 0.02 (-0.49 to 0.53, p=0.939) | 0.07 (-0.29 to 0.42, p=0.717) |
|  |  | + covariates | 0.17 (-0.43 to 0.76, p=0.580) | 0.09 (-0.47 to 0.66, p=0.743) | 0.15 (-0.25 to 0.54, p=0.462) |
|  | **PM_2.5_, peak** | crude | -0.01 (-0.39 to 0.37, p=0.955) | 0.01 (-0.36 to 0.37, p=0.977) | 0.13 (-0.13 to 0.38, p=0.329) |
|  |  | + covariates | 0.16 (-0.29 to 0.61, p=0.480) | 0.08 (-0.33 to 0.50, p=0.692) | 0.25 (-0.04 to 0.54, p=0.091) |
|  | **PM_2.5-10_, mean** | crude | 0.30 (-0.10 to 0.69, p=0.139) | 0.09 (-0.27 to 0.45, p=0.623) | 0.09 (-0.16 to 0.35, p=0.463) |
|  |  | + covariates | 0.33 (-0.11 to 0.77, p=0.138) | 0.11 (-0.28 to 0.50, p=0.580) | 0.12 (-0.16 to 0.39, p=0.408) |
|  | **PM_2.5-10_, peak** | crude | 0.24 (-0.10 to 0.59, p=0.164) | 0.18 (-0.13 to 0.49, p=0.251) | 0.10 (-0.12 to 0.32, p=0.384) |
|  |  | + covariates | 0.23 (-0.17 to 0.64, p=0.249) | 0.17 (-0.18 to 0.53, p=0.339) | 0.14 (-0.11 to 0.39, p=0.281) |
|  | **Exposure Group (ref=reference population)** | crude | -0.28 (-1.01 to 0.46, p=0.454) | -0.11 (-0.81 to 0.58, p=0.747) | -0.20 (-0.70 to 0.30, p=0.433) |
|  |  | + covariates | 0.06 (-0.88 to 1.01, p=0.897) | 0.04 (-0.84 to 0.91, p=0.935) | -0.12 (-0.75 to 0.51, p=0.701) |
|  |  | + exposure Group* PM1, mean | -0.45 (-1.75 to 0.84, p=0.488) | 0.47 (-0.73 to 1.66, p=0.441) | -0.03 (-0.90 to 0.84, p=0.943) |
|  |  | + exposure Group* PM1, peak | -0.45 (-1.47 to 0.56, p=0.376) | 0.33 (-0.60 to 1.27, p=0.480) | -0.17 (-0.83 to 0.49, p=0.605) |
|  |  | + exposure Group* PM2.5, mean | 0.07 (-1.55 to 1.70, p=0.930) | 1.14 (-0.36 to 2.63, p=0.133) | -0.06 (-1.15 to 1.04, p=0.918) |
|  |  | + exposure Group* PM2.5, peak | -0.09 (-1.14 to 0.97, p=0.873) | 1.08 (0.07 to 2.08, p=0.036) | -0.27 (-0.97 to 0.43, p=0.442) |
|  |  | + exposure Group*PM_2.5-10_, mean | 0.17 (-0.90 to 1.23, p=0.759) | 0.53 (-0.38 to 1.45, p=0.247) | -0.17 (-0.86 to 0.52, p=0.622) |
|  |  | + exposure Group* PM_2.5-10_, peak | 0.20 (-0.66 to 1.06, p=0.648) | 0.61 (-0.17 to 1.38, p=0.122) | -0.31 (-0.85 to 0.24, p=0.268) |
| Pre-Shift Exposures | |  |  |  |  |
|  | **Worked yesterday (day before the PF test) , (ref=No)** | crude | -0.34 (-1.17 to 0.50, p=0.428) | -0.77 (-1.55 to 0.02, p=0.055) | 0.16 (-0.41 to 0.74, p=0.571) |
|  |  | + covariates | -0.19 (-1.20 to 0.82, p=0.712) | -1.22 (-2.18 to -0.27, p=0.013) | 0.57 (-0.13 to 1.27, p=0.111) |
|  |  | + worked yesterday*exposure group | -2.01 (-4.02 to 0.01, p=0.051) | -1.79 (-3.57 to -0.02, p=0.048) | -0.26 (-1.58 to 1.06, p=0.696) |
|  | **Worked prior to the pre-shift PF test (same day), (ref=No)** | crude | -0.21 (-0.94 to 0.52, p=0.569) | -0.24 (-0.92 to 0.44, p=0.488) | -0.32 (-0.81 to 0.17, p=0.193) |
|  |  | + covariates | -0.07 (-0.88 to 0.74, p=0.861) | 0.05 (-0.70 to 0.80, p=0.892) | -0.35 (-0.89 to 0.18, p=0.191) |
|  |  | + worked prior*exposure group | 0.48 (-1.29 to 2.26, p=0.591) | -0.34 (-1.99 to 1.30, p=0.682) | -0.19 (-1.37 to 0.99, p=0.747) |

^a^ Number of sessions with a valid pre and post-shift FEV1 and personal PM monitoring data = 151; ^b^ Number of participants with a valid pre and post-shift FVC and personal PM monitoring data = 123; ^c^ Number of participants with a valid pre and post-shift Ratio and personal PM monitoring data = 118; ^d^ Covariates in the adjusted models include age, height, use of cigarettes during the shift, wave of data collection and day of week.

**Supplemental Table 3:** Stratified analysis of the association between pulmonary function (cross-shift and percent predicted) and working "yesterday" (the day before pulmonary function testing) in E-waste workers (n=90) and a reference population (n=64) enrolled in the GeoHealth Cohort, Accra, Ghana, 2017-2018.

|  |  | E-Waste Workers (n=90 work-shifts) | | | Reference Population (n=64 work shifts) | | |
| --- | --- | --- | --- | --- | --- | --- | --- |
|  |  | **ΔFEV1 (% change / hour)** | **FEV1 pre-shift % predicted** | **FEV1 post-shift % predicted** | **ΔFEV1 (% change / hour)** | **FEV1 pre-shift % predicted** | **FEV1 post-shift % predicted** |
| Work Yesterday (ref=No) | | RR (95%CI) | RR (95%CI) | RR (95%CI) | RR (95%CI) | RR (95%CI) | RR (95%CI) |
|  | crude | -1.31 (-3.03, 0.42) | -5.61 (-14.26, 3.03) | -8.95 (-17.99, 0.09) | 0.38 (-0.36, 1.13) | 1.62 (-3.57, 6.80) | 3.05 (-2.40, 8.50) |
|  | + covariates^a^ | -1.19 (-3.07, 0.69) | -9.21 (-18.04, -0.38) | -12.07 (-21.36, -2.78) | 0.28 (-0.62, 1.18) | 0.68 (-6.56, 7.92) | 2.24 (-5.30, 9.78) |
|  |  |  |  |  |  |  |  |
|  |  | **ΔFVC (% change / hour)** | **FVC pre-shift % predicted** | **FVC post-shift % predicted** | **ΔFVC (% change / hour)** | **FVC pre-shift % predicted** | **FVC post-shift % predicted** |
| Work Yesterday (ref=No) | | RR (95%CI) | RR (95%CI) | RR (95%CI) | RR (95%CI) | RR (95%CI) | RR (95%CI) |
|  | crude | -1.69 (-3.15, -0.23) | 0.96 (-8.05, 9.98) | -4.74 (-15.09, 5.60) | -0.16 (-0.97, 0.65) | 5.03 (-1.56, 11.61) | 4.07 (-1.56, 9.70) |
|  | + covariates^a^ | -2.43 (-4.04, -0.81) | 0.70 (-8.81, 10.21) | -6.49 (-17.37, 4.38) | -0.25 (-1.30, 0.79) | 4.32 (-5.18, 13.82) | 4.18 (-3.12, 11.49) |

^a^ Covariates in the adjusted models include age, height, use of cigarettes during the shift, wave of data collection and day of week.

**Supplemental Table 4:** Incident respiratory symptoms reported among e-waste and reference population study participants (N=156 work shifts; 92 E-waste worker shifts and 47 reference population shifts) enrolled in the GeoHealth cohort, Accra, Ghana, 2017-2018.

| Incident symptom | E-waste worker | Reference population | p-value |
| --- | --- | --- | --- |
| Irritation or burning of the eyes, nose or throat (%) | 13.0 | 14.1 | 1 |
| Chest tightness or a sensation of a band around the chest (%) | 9.8 | 3.1 | 0.2 |
| Cough (%) | 17.4 | 11.1 | 0.36 |
| Shortness of breath, difficulty catching your breath, or a smothering feeling (%) | 9.8 | 6.2 | 0.56 |
| Wheezing or whistling sound in your chest apart from colds (%) | 16.3 | 7.8 | 0.15 |
